# Detailed severity assessment of Cincinnati Prehospital Stroke Scale to detect large vessel occlusion in acute ischemic stroke

**DOI:** 10.1186/s12873-020-00360-9

**Published:** 2020-08-24

**Authors:** Gabor Tarkanyi, Peter Csecsei, Istvan Szegedi, Evelin Feher, Adam Annus, Tihamer Molnar, Laszlo Szapary

**Affiliations:** 1 Department of Neurology, University of Pecs, 13 Ifjusag utja, Pecs, 7624 Hungary; 2 Department of Neurology, University of Debrecen, Debrecen, Hungary; 3 Department of Neurology, University of Szeged, Szeged, Hungary; 4 Department of Anaesthesiology and Intensive Therapy, University of Pecs, Pecs, Hungary

**Keywords:** Acute stroke, Large vessel occlusion, Stroke scales, Prehospital, Emergency medicine, Neurology

## Abstract

**Background:**

Selecting stroke patients with large vessel occlusion (LVO) based on prehospital stroke scales could provide a faster triage and transportation to a comprehensive stroke centre resulting a favourable outcome. We aimed here to explore the detailed severity assessment of Cincinnati Prehospital Stroke Scale (CPSS) to improve its ability to detect LVO in acute ischemic stroke (AIS) patients.

**Methods:**

A cross-sectional analysis was performed in a prospectively collected registry of consecutive patients with first ever AIS admitted within 6 h after symptom onset. On admission stroke severity was assessed using the National Institutes of Health Stroke Scale (NIHSS) and the presence of LVO was confirmed by computed tomography angiography (CTA) as an endpoint. A detailed version of CPSS (d-CPSS) was designed based on the severity assessment of CPSS items derived from NIHSS. The ability of this scale to confirm an LVO was compared to CPSS and NIHSS respectively.

**Results:**

Using a ROC analysis, the AUC value of d-CPSS was significantly higher compared to the AUC value of CPSS itself (0.788 vs. 0.633, *p* < 0.001) and very similar to the AUC of NIHSS (0.795, *p* = 0.510). An optimal cut-off score was found as d-CPSS≥5 to discriminate the presence of LVO (sensitivity: 69.9%, specificity: 75.2%).

**Conclusion:**

A detailed severity assessment of CPSS items (upper extremity weakness, facial palsy and speech disturbance) could significantly increase the ability of CPSS to discriminate the presence of LVO in AIS patients.

## Background

Endovascular thrombectomy (EVT) is effective to treat patients with acute ischemic stroke (AIS) caused by large vessel occlusion (LVO), which occurs in 20–40% of cases [1, 2]. There is a growing need for simple diagnostic methods that can detect these patients early on. A reliable LVO detection tool could be useful for emergency medical services (EMS) to select patients with a high likelihood of LVO, as these patients may benefit from a direct transportation to an EVT capable comprehensive stroke centre (CSC) [3].

Cincinnati Prehospital Stroke Scale (CPSS) is a simple, three item scale, widely used by EMS. It is easy and quick to learn or perform and has good ability to identify potential stroke patients. Nonetheless, it only has moderate ability to detect AIS patients with LVO, however, important aspect is that CPSS only tests for the presence of three symptoms (facial palsy, upper extremity weakness and speech disturbance), but do not assess the severity of them [4–6]. The aim of our study was to examine whether the detailed severity assessment of these items can improve the overall ability of CPSS to detect LVO in AIS patients.

## Methods

### Study population

We have performed a cross-sectional analysis based on a prospectively collected registry of consecutive patients with first ever AIS, who were admitted up to 6 h after symptom onset to the CSC of three university hospitals between November 2017 and July 2019 (more information on this registry is presented in the Supplementary material). Demographic data, vascular risk factors, baseline clinical variables and time from onset to first assessment in the emergency room were recorded on admission, along with detailed evaluation of the National Institutes of Health Stroke Scale (NIHSS). Our outcome of interest was the presence of LVO on the on admission computed tomography angiography (CTA) scan, evaluated by trained neuroradiologists as a standard of care. The results were subsequently checked by one of the authors (PC or LS) who were blinded to the clinical parameters and stroke severity. In case of disagreement final decision was made after personal communication. NIHSS was routinely assessed before CTA was performed. According to *Rennert* et al. [7] unilateral occlusion of the internal carotid artery (ICA), occlusion in the M1, M2 or M3 segment of the middle cerebral artery (MCA), occlusion of the anterior cerebral artery (ACA), vertebral artery (VA), basilar artery (BA) and posterior cerebral artery (PCA) occlusions were considered. Based on the 2019 update of the 2018 guidelines for the early management of AIS by the American Heart Association and the American Stroke Association we have created three groups of LVO patients. In the first group we have included patients with ICA or M1 occlusions as there is a strong (class Ia) recommendation to consider EVT in these patients. In the second group patients with LVO in the more distal segments of the anterior vascular territory (M2, M3 segments of MCA, ACA) were included. The third group included those with LVO in the posterior circulation (VA, BA or PCA). In these cases, the benefit of EVT is uncertain, however it should be considered on a case-by-case basis (recommendation class IIb and IIc respectively) [8, 9]. Patients who did not have CTA scan on admission were excluded.

### Scale design

We derived CPSS from four items of NIHSS (item 4: facial palsy, item 5: unilateral upper extremity weakness, item 9: language and item 10: dysarthria), according to *Kothari* et al. [4] we have combined NIHSS items 9 and 10 to get the speech item of CPSS. We designed a detailed version of CPSS (d-CPSS) derived from the same NIHSS items, but without being converted to bivariate as in CPSS. Detailed scoring criteria are shown in Table 1. The ability of d-CPSS to discriminate an LVO was compared to the ability of CPSS and NIHSS.

**Table 1:** Detailed scoring of CPSS and d-CPSS compared to NIHSS scores

Severity of symptoms	CPSS score	d-CPSS score	NIHSS source item and score	
ARM			Item 5: arm motor drift	
No drift for 10 s	0	0	0	
Drift, but does not hit bed	1	1	1	
Some effort against gravity	1	2	2	
No effort against gravity	1	3	3	
No movement	1	4	4	
FACIAL PALSY			Item 4: facial palsy	
Normal symmetry	0	0	0	
Minor paralysis	1	1	1	
Partial paralysis	1	2	2	
Complete paralysis	1	3	3	
SPEECH			Item 9: aphasia	Item 10: dysarthria
Normal	0	0	0	0
Mild/moderate aphasia or dysarthria	1	1	1	1
Severe aphasia or dysarthria	1	2	2	2
Global aphasia or anarthic or mute	1	3	3	2
TOTAL	0–3	0–10		

Abbreviation: *CPSS* Cincinnati Prehospital Stroke Scale; *d-CPSS* Detailed CPSS; *NIHSS* National Institutes of Health Stroke Scale

### Statistical analysis

Data analysis was performed using SPSS (version 26.0, IBM, New York). Continuous variables were presented as mean and standard deviation (SD) or as median and interquartile range (IQR) where appropriate. Categorical variables were presented as counts and percentages. Comparison of continuous variables were performed using *t* test or *Mann-Whitney U* test. Normality was assessed using the *Shapiro-Wilk* test and visually, based on Q-Q plots and histograms. *Kruskal-Wallis* test was used to compare stroke scale scores between multiple groups. Categorical data were compared using the *Pearson X*^*2*^ test. Binary logistic regression with enter method was used to assess associations between baseline clinical variables and the presence of LVO. Adjustment was made for potential confounders, variables with *P* < 0.1 in the univariable analysis were entered to the multivariable logistic regression model. Stroke scales and symptoms were entered in separate models because of multicollinearity. The ability of scales to detect the presence of LVO and optimal cut-off points was assessed using the receiver operating characteristic (ROC) analysis. Area under the curve (AUC) was calculated for each scale and *z* test was used for comparison. Sensitivity (SN), specificity (SP), positive and negative predictive values and accuracy were calculated for different cut-off values. Where appropriate 95% confidence intervals (CI) were presented. A *P* value < 0.05 was considered statistically significant.

## Results

During the study period 528 patients were screened, 421 (79.7%) of whom underwent CTA imaging. The mean age of the study cohort was 67.2 ± 13.2 years (48.7% female), 183 patients had LVO (43.5%). Baseline demographics and clinical factors of the two studied groups (according to the presence of LVO) are shown in Table 2. On admission CPSS, d-CPSS and NIHSS scores were significantly higher in those with LVO. The frequency of upper extremity weakness (92.3% vs. 71.8%, *p* < 0.001) and facial palsy (85.8% vs. 69.8%, *p* < 0.001) were higher among LVO patients, but there was no significant difference in the presence of speech disturbance between the groups (77.0% vs. 74.5%, *p* = 0.408). After adjustment for potential confounders (onset-to-assessment time, systolic and diastolic blood pressure, the presence of atrial fibrillation, coronary artery disease and chronic heart failure), significant associations were observed between LVO and: (i) known atrial fibrillation (AF) (OR: 2.564, *p* < 0.001); (ii) systolic blood pressure (SBP) on admission (OR: 0.904 per 10 mmHg increase, *p* = 0.046); (iii) the presence of upper extremity weakness (OR: 5.370, *p* < 0.001); and (iv) the presence of facial palsy (OR: 3.107, *p* < 0.001). Increasing severity of all three symptoms examined in d-CPSS were independently associated with higher odds of LVO presence. Higher CPSS, d-CPSS and NIHSS scores were also associated with increased odds of LVO (detailed results are presented in **Table** **S1** in the Supplementary material).

**Table 2:** Demography and clinical characteristics of the cohort according to the presence of LVO

	LVO present (*N* = 183)	LVO absent (*N* = 238)	*P* value
Age, years, median (IQR)	67 (60–78)	69 (58.75–76.25)	0.652
Gender, female, % (n)	52.5 (96)	45.8 (109)	0.175
NIHSS score, median (IQR)	11 (6–16)	6 (4–9)	**< 0.001**
CPSS score, median (IQR)	3 (2–3)	2 (2–3)	**< 0.001**
d-CPSS score, median (IQR)	5 (3–7)	3 (2–4.25)	**< 0.001**
Onset to ER assessment time, min, median (IQR)	80 (58–121.25)	92 (58.75–137.25)	0.053
On admission SBP, mmHg, mean (SD)	159.0 (30.3)	167.8 (29.9)	**0.003**
On admission DBP, mmHg, mean (SD)	88.2 (16.0)	91.2 (17.1)	0.066
Smoking, % (n), 51 missing	37.0 (57)	31.5 (68)	0.267
Hypertension, % (n), 15 missing	79.5 (140)	79.6 (183)	0.996
Diabetes mellitus, % (n), 19 missing	19.4 (34)	26.0 (59)	0.122
Hyperlipidaemia, % (n), 37 missing	55.7 (93)	55.3 (120)	0.939
Atrial fibrillation, % (n), 23 missing	35.8 (62)	16.9 (38)	**< 0.001**
Coronary artery disease, % (n), 29 missing	25.9 (45)	17.4 (38)	**0.042**
Chronic heart failure, % (n), 25 missing	14.4 (25)	8.1 (18)	**0.047**

Abbreviation: *LVO* Large vessel occlusion; *NIHSS* National Institutes of Health Stroke Scale; *IQR* Interquartile range; *CPSS* Cincinnati Prehospital Stroke Scale; *d-CPSS* Detailed CPSS; *ER* Emergency room; *SBP* Systolic blood pressure; *DBP* Diastolic blood pressure; *SD* Standard deviation

**Fig. 1:**
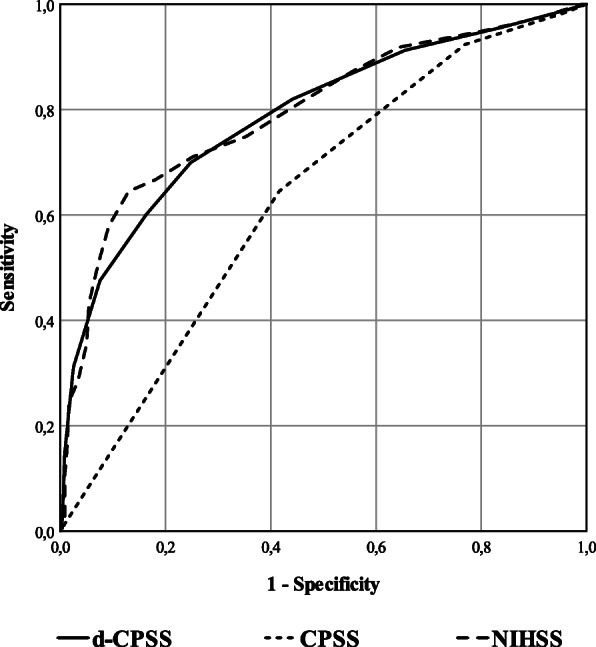
Receiver operating characteristic curves describing the capability of investigated scales to confirm a large vessel occlusion in acute ischemic stroke: Cincinnati Prehospital Stroke Scale (CPSS), detailed CPSS (d-CPSS) and National Institutes of Health Stroke Scale (NIHSS)

Using a ROC analysis, the AUC value of d-CPSS was significantly higher compared to the AUC value of CPSS itself (0.788, 95% CI: 0.743 to 0.832 vs. 0.633, 95% CI: 0.580 to 0.686; *p* < 0.001). The AUC for NIHSS was 0.795 (95% CI: 0.751 to 0.839), which was not significantly different from the AUC for d-CPSS (*p* = 0.510). ROC curves are presented in Fig. 1. The optimal cut-off scores to discriminate an LVO were CPSS = 3 (SN: 64.5%, SP: 58.4%), d-CPSS≥5 (SN: 69.9%, SP: 75.2%) and NIHSS≥11 (SN: 64,5%, SP: 87.0%) respectively (**Table** **S2** in the Supplementary material).

**Fig. 2:**
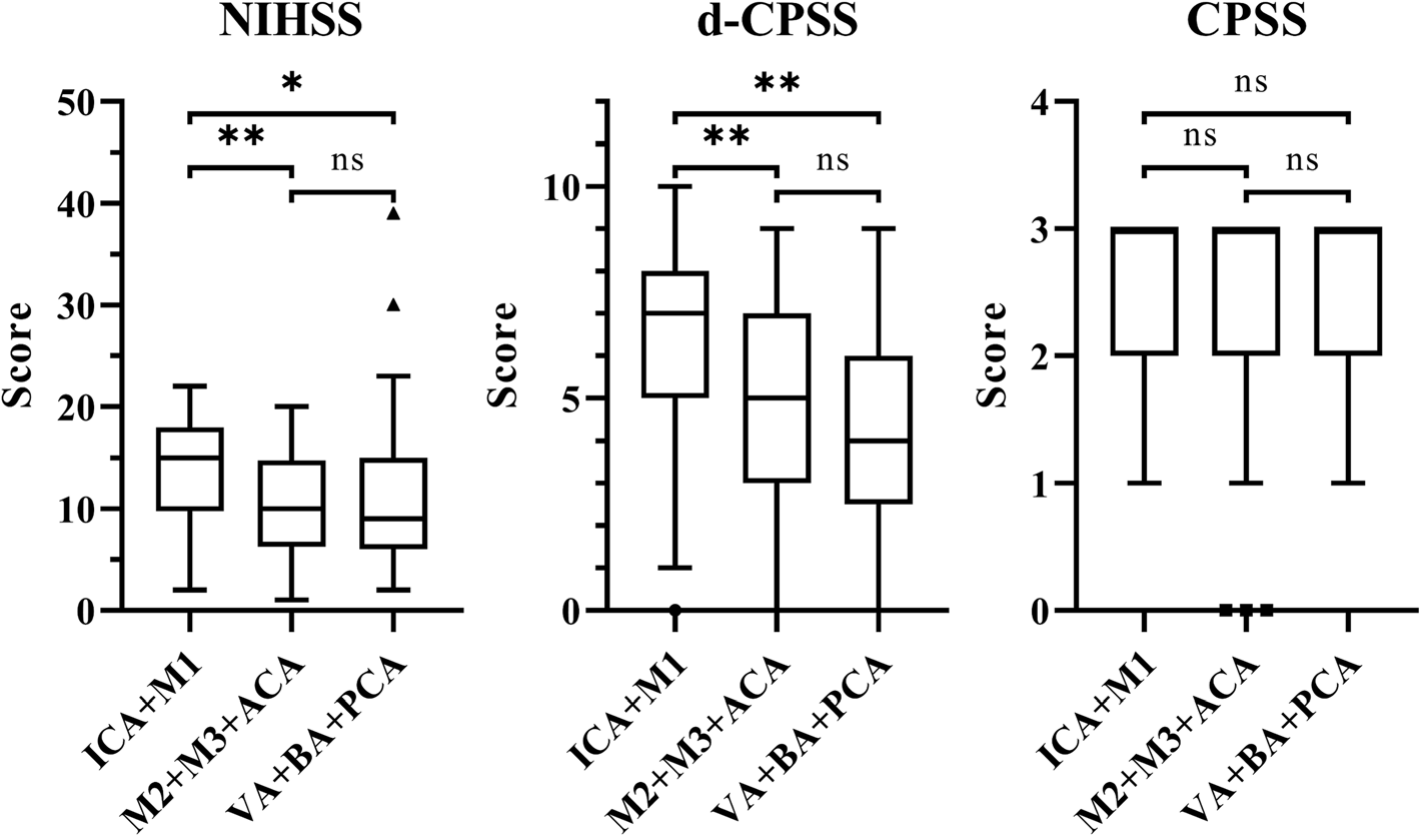
Differences in National Institutes of Health Stroke Scale (NIHSS), detailed CPSS (d-CPSS) and Cincinnati Prehospital Stroke Scale (CPSS) scores between groups according to the location of large vessel occlusion (LVO): proximal LVO in the anterior vascular territory (internal carotid artery [ICA] and M1 segment of the middle cerebral artery [MCA]; *n* = 118), distal LVO in the anterior circulation (M2 or M3 segments of MCA and anterior cerebral artery [ACA]; *n* = 48) and posterior circulation LVO (vertebral artery [VA], basilar artery [BA] and posterior cerebral artery [PCA]; *n* = 17). Boxes, 25 to 75% interquartile range; central horizontal bars, median; outer horizontal bars, minimum and maximum values excluding outliers (triangle, dot or square icons). Abbreviation: ns, not significant; *, *p* ≤ 0.05; **, *p* ≤ 0.001

Median NIHSS and d-CPSS scores tended to be higher in patients with LVO in the ICA or M1 segment of MCA compared to those with LVO in the more distal segments of the anterior vascular territory (M2, M3, ACA) (NIHSS: 15 vs. 10, *p* < 0.001; d-CPSS: 7 vs. 5, *p* = 0.001). Patients with ICA or M1 occlusions had higher median NIHSS and d-CPSS scores than patients with posterior circulation LVO (VA, BA, PCA) (NIHSS: 15 vs. 9, *p* < 0.047; d-CPSS: 7 vs. 4, *p* = 0.001). No significant difference in NIHSS and d-CPSS scores were found between the distal anterior territory LVO and posterior LVO groups (*p* = 0.697 and 0.274 respectively). No differences were recorded in CPSS scores between these groups (median score: 3 respectively; *p* = 0.783) (see Fig. 2).

## Discussion

The main finding of our study is that detailed severity assessment of CPSS items (upper extremity weakness, facial palsy and speech disturbance) could significantly increase the ability of CPSS to discriminate the presence of LVO in AIS patients.

Currently NIHSS is the gold-standard of stroke severity assessment and it has good ability to detect LVO [10]. However, its complexity, time-consuming nature and the need for a special training can make its application in emergency situations or prehospital environment challenging [11]. Our results suggest that a detailed evaluation of CPSS may have similar capabilities as NIHSS to predict the presence of LVO, nonetheless, both NIHSS and d-CPSS still misdiagnose a significant proportion of stroke patients.

The definition of LVO is heterogenous among studies according to different diagnostic and therapeutic approaches [7]. Endovascular thrombectomy is primarily recommended within 6 h from symptom onset in cases of ICA or M1 occlusions, however more distal and posterior occlusions might also be treatable using EVT on a case-by-case basis [8]. Perhaps the main aim of prehospital LVO detection is to identify patients who should undergo adequate EVT eligibility screening early on, therefore the identification of every type of LVO may be useful in this regard.

Our findings are highlighting that stroke severity may related to the location of LVO as NIHSS and d-CPSS scores tended to be the higher in cases of proximal occlusions (ICA or M1) than in those with more distal or posterior occlusions. This result suggests that it may be worth considering proximal LVO in patients with high NIHSS or d-CPSS scores, but it should be noted that posterior LVO may also cause severe strokes, which is also shown by our results (Fig. 2). However, this tendency is not noticeable for CPSS, which points out the benefit of detailed severity analysis in d-CPSS.

Over the past few years, attempts have been made to develop new, shorter and modified LVO detection scales in order to fit them for prehospital use, but only few have been examined extensively yet and only a minority of them have been implemented into the practice of EMS [12]. Since CPSS is one of the most widely used and well-established scales in the field of stroke assessment, it would be obvious to optimize this scale for early LVO detection.

Our results are consistent with previous studies suggesting that certain baseline variables (e.g. known AF, SBP on admission) and the presence of certain symptoms (especially aphasia, neglect and hemiparesis) are related to the presence of LVO [13, 14]. The presence of speech disturbance is not, but its severity was associated with LVO in our study, which highlights how severity assessment may improve stroke scales. Weighting of scale items or adding anamnestic data (such as history of AF) to stroke scales could improve their ability to predict LVO in AIS [14, 15].

Based on previous result and the findings of our study, we think that future studies should focus on optimizing existing stroke scales to LVO detection, instead of developing new ones. More detailed severity assessment or proper weighting of symptoms could be a good perspective and adding items to scales that are strongly associated with LVO could also be beneficial and should be considered. Prehospital prospective validation of these scales and comparison of their predictive power should also be the scope of further studies. Furthermore, the impact of such scales on prehospital pathways in cases of different likelihoods of LVO should also be clarified. Another interesting scope of future stroke scale studies could be not only the detection of LVO but the early recognition of patients potentially eligible for thrombectomy taking other indication criteria (Alberta stroke program early CT score, age, pre-stroke modified Rankin Scale score etc.) into consideration.

The retrospective analysis of prospectively collected data is the main limitation of our study. Besides, we only examined patients with AIS, and we did not have data on patients with haemorrhagic stroke and stroke-mimics. A significant proportion of screened AIS patients did not have CTA imaging, mainly due to minor symptoms (**Table** **S3** in the Supplementary material), which may have caused selection bias. The assessment of CTA scans was performed by neuroradiologists as a standard of care, however no inter-rater reliability test was performed which might have led to diagnostic bias. It is important to highlight that we did not prospectively validate d-CPSS in this study, however we intend to do so in the future, with the abovementioned considerations in mind.

## Conclusions

In conclusion, we can say that detailed severity assessment of symptoms can improve the ability of CPSS to detect LVO in AIS, while remaining simple to perform. Despite the remarkable number of stroke scales developed, future studies should focus on optimizing existing well-established scales, aiming to provide a faster triage and therapeutic intervention for AIS patients with LVO.

## Supplementary information

**Additional file 1: Table S1.** Associations between baseline characteristics and LVO. **Table S2.** Diagnostic performance of investigated scales according to different cut-off values. **Table S3.** Baseline characteristics of the included and excluded patients.

## Data Availability

The datasets used and/or analysed during the current study are available from the corresponding author on reasonable request.

## Abbreviations

EVT: Endovascular thrombectomy
LVO: Large vessel occlusion
AIS: Acute ischemic stroke
EMS: Emergency medical service
CSC: Comprehensive stroke centre
CPSS: Cincinnati prehospital stroke scale
NIHSS: National institutes of health stroke scale
CTA: Computed tomography angiography
ICA: Internal carotid artery
MCA: Middle cerebral artery
ACA: Anterior cerebral artery
VA: Vertebral artery
BA: Basilar artery
PCA: Posterior cerebral artery
d-CPSS: Detailed cincinnati prehospital stroke scale
SD: Standard deviation
IQR: Interquartile range
ROC: Receiver operating characteristic
AUC: Area under the curve
SN: Sensitivity
SP: Specificity
CI: Confidence interval
OR: Odds ratio
AF: Atrial fibrillation
SBP: Systolic blood pressure

## References

[1] Goyal M, Menon BK, van Zwam WH, Dippel DWJ, Mitchell PJ, Demchuk AM (2016). Endovascular thrombectomy after large-vessel ischaemic stroke: a meta-analysis of individual patient data from five randomised trials. Lancet.

[2] Lakomkin N, Dhamoon M, Carroll K, Singh IP, Tuhrim S, Lee J (2019). Prevalence of large vessel occlusion in patients presenting with acute ischemic stroke: a 10-year systematic review of the literature. J Neurointerv Surg.

[3] Venema E, Lingsma HF, Chalos V, Mulder MJHL, Lahr MMH, van der Lugt A (2019). Personalized prehospital triage in acute ischemic stroke. Stroke.

[4] Kothari R, Hall K, Brott T, Broderick J (1997). Early stroke recognition: developing an out-of-hospital NIH stroke scale. Acad Emerg Med.

[5] Richards CT, Huebinger R, Tataris KL, Weber JM, Eggers L, Markul E (2018). Cincinnati prehospital stroke scale can identify large vessel occlusion stroke. Prehosp Emerg Care.

[6] Antipova D, Eadie L, Macaden A, Wilson P (2019). Diagnostic accuracy of clinical tools for assessment of acute stroke: a systematic review. BMC Emerg Med.

[7] Rennert RC, Wali AR, Steinberg JA (2019). Epidemiology, natural history, and clinical presentation of large vessel ischemic stroke. Neurosurgery.

[8] Powers WJ, Rabinstein AA, Ackerson T (2019). Guidelines for the early management of patients with acute ischemic stroke: 2019 update to the 2018 guidelines for the early management of acute ischemic stroke: a guideline for healthcare professionals from the American Heart Association/American Stroke Association. Stroke.

[9] Uno J, Kameda K, Otsuji R (2018). Mechanical thrombectomy for acute anterior cerebral artery occlusion. World Neurosurg.

[10] Smith EE, Kent DM, Bulsara KR, Leung LY, Lichtman DH, Reeves MJ (2018). Accuracy of prediction instruments for diagnosing large vessel occlusion in individuals with suspected stroke: a systematic review for the 2018 guidelines for the early management of patients with acute ischemic stroke. Stroke.

[11] Hov MR, Røislien J, Lindner T, Zakariassen E, Bache KCG, Solyga VM, Russell D, Lund CG (2019). Stroke severity quantification by critical care physicians in a mobile stroke unit. Eur J Emerg Med.

[12] Vidale S, Agostoni E (2018). Prehospital stroke scales and large vessel occlusion: a systematic review. Acta Neur Scand.

[13] Inoue M, Noda R, Yamaguchi S, Tamai Y, Miyahara M, Yanagisawa S (2018). Specific factors to predict large-vessel occlusion in acute stroke patients. J Stroke Cerebrovasc Dis.

[14] Beume LA, Hieber M, Kaller CP, Nitschke K, Bardutzky J, Urbach H (2018). Large vessel occlusion in acute stroke. Stroke.

[15] Narwal P, Chang AD, Grory BM, Jayaraman M, Madsen T, Paolucci G (2019). The addition of atrial fibrillation to the Los Angeles motor scale may improve prediction of large vessel occlusion. J Neuroimaging.

