# Supplementary material for: Detailed severity assessment of Cincinnati Prehospital Stroke Scale to detect large vessel occlusion in acute ischemic stroke
Source: BMC Emerg Med. 2020 Aug 24;20:64. doi: 10.1186/s12873-020-00360-9 (PMC7446055; doi:10.1186/s12873-020-00360-9)
Supplement: Supplementary file 1 — Additional file 1: Table S1. Associations between baseline characteristics and LVO. Table S2. Diagnostic performance of investigated scales according to different cut-off values. Table S3. Baseline characteristics of the included and excluded patients. [file 12873_2020_360_MOESM1_ESM.docx]

**Supplementary material**

**Detailed severity assessment of Cincinnati Prehospital Stroke Scale to detect large vessel occlusion in acute ischemic stroke**

Tarkanyi G, Csecsei P, Szegedi I, Feher E, Annus A, Molnar T. Szapary L.

Study population, STAY ALIVE acute stroke registry

In this study we used data from the STAY ALIVE acute stroke registry, which is a part of GINOP 2.3.2-15-2016-00048 Stay Alive. This registry is a prospectively collected, ongoing, national, hospital-based, multicentre database of acute ischemic stroke patients including comprehensive stroke centres of three university hospitals in Hungary (University of Debrecen, University of Szeged and University of Pécs). Patients who are admitted to one of these stroke centres due to acute ischemic stroke are prospectively screened and enrolled to the registry. Participation is voluntary and written informed consent is obtained from each patient. Detailed data on medical history, on admission parameters, imaging results, interventions, medical investigations, etiology and follow-up data are collected by clinical research administrators and medical doctors. Data are recorded on an electronic case report form (eCRF) and subsequently checked and approved by an assigned trained neurologist and by the chief research administrator. Final approval is made by the head of each department who are also the guarantors. Detailed information can be found at https://tm-centre.org/en/registries/stroke-registry/.

**Table S1** Associations between baseline characteristics and LVO

|  | Univariable analysis (95% CI) | P value | Multivariable analysis (95% CI) | P value |
| --- | --- | --- | --- | --- |
| Age, years | 1.005 (0.991-1.020) | 0.485 |  |  |
| Gender, female | 1.306 (0.882-1.922) | 0.176 |  |  |
| NIHSS score, 1-point increase | 1.255 (1.196-1.317) | <0.001 | 1.273 (1.205-1.345) | **<0.001** |
| CPSS score, 1-point increase | 1.970 (1.504-2.580) | <0.001 | 2.123 (1.567-2.875) | **<0.001** |
| d-CPSS score, 1-point increase | 1.651 (1.485-1.836) | <0.001 | 1.695 (1.506-1.906) | **<0.001** |
| Onset-to-assessment time, 1 min. increase | 0.997 (0.993-1.000) | **0.057*** | 0.997 (0.993-1.001) | 0.190 |
| On admission SBP, 10 mmHg increase | 0.907 (0.849-0.969) | **0.004*** | 0.904 (0.818-0.998) | **0.046** |
| On admission DBP, 10 mmHg increase | 0.895 (0.795-1.008) | **0.067*** | 1.040 (0.873-1.240) | 0.661 |
| Smoking | 1.279 (0.828-1.976) | 0.268 |  |  |
| Hypertension | 0.999 (0.614-1.625) | 0.996 |  |  |
| Diabetes mellitus | 0.687 (0.426-1.107) | 0.123 |  |  |
| Hyperlipidaemia | 1.016 (0.677-1.525) | 0.939 |  |  |
| Atrial fibrillation | 2.749 (1.723-4.385) | **<0.001*** | 2.654 (1.620-4.348) | **<0.001** |
| Coronary artery disease | 1.652 (1.015-2.690) | **0.043*** | 1.478 (0.868-2.519) | 0.151 |
| Chronic heart failure | 1.902 (1.001-3.612) | **0.050*** | 1.238 (0.599-2.559) | 0.565 |
| Presence of upper extremity weakness | 4.730 (2.560-8.739) | <0.001 | 5.370 (2.771-10.408) | **<0.001** |
| Presence of facial palsy | 2.725 (1.657-4.481) | <0.001 | 3.107 (1.778-5.429) | **<0.001** |
| Presence of speech disturbance | 1.209 (0.771-1.894) | 0.408 | 1.166 (0.710-1.914) | 0.545 |
| Severity of upper extremity weakness, 1-point increase | 2.057 (1.753-2.413) | <0.001 | 2.045 (1.721-2.430) | **<0.001** |
| Severity of facial palsy, 1-point increase | 1.913 (1.513-2.419) | <0.001 | 2.133 (1.628-2.795) | **<0.001** |
| Severity of speech disturbance, 1-point increase | 2.090 (1.677-2.605) | <0.001 | 2.299 (1.789-2.953) | **<0.001** |

Abbreviation: LVO, large vessel occlusion; 95% CI, 95% confidence interval; NIHSS, National Institutes of Health Stroke Scale; IQR, interquartile range; CPSS, Cincinnati Prehospital Stroke Scale; d-CPSS, detailed CPSS; SBP, systolic blood pressure; DBP, diastolic blood pressure.

***** Variables included in the multivariable analysis.

**Table S2** Diagnostic performance of investigated scales according to different cut-off values

| **Scale,**  **cut-off point** | **Sensitivity**  **(95% CI)** | **Specificity**  **(95% CI)** | **Positive predictive value (95% CI)** | **Negative predictive value (95% CI)** | **Accuracy (95% CI)** |
| --- | --- | --- | --- | --- | --- |
| CPSS ≥1 | 98.4  (95.3 to 99.7) | 4.2  (2.0 to 7.6) | 44.1  (43.3 to 44.9) | 76.9  (48.2 to 92.3) | 45.1  (40.3 to 50.0) |
| CPSS ≥2 | 92.4  (87.5 to 95.8) | 23.1  (17.9 to 30.0) | 48.0  (46.0 to 50.0) | 79.7  (69.3 to 87.2) | 53.2  (48.3 to 58.1) |
| CPSS =3 | 64.5  (57.1 to 71.4) | 58.4  (51.9 to 64.7) | 54.4  (49.8 to 58.9) | 68.1  (63.1 to 72.8) | 61.1  (56.2 to 65.3) |
| d-CPSS ≥1 | 98.9  (96.1 to 99.9) | 3.8  (1.7 to 7.1) | 44.2  (43.4 to 44.9) | 81.8  (49.6 to 95.4) | 45.1  (40.3 to 50.0) |
| d-CPSS ≥2 | 95.6  (91.2 to 98.9) | 16.0  (11.6 to 21.3) | 46.7  (45.1 to 48.3) | 82.6  (69.4 to 90.9) | 50.6  (45.7 to 55.5) |
| d-CPSS ≥3 | 91.3  (86.2 to 94.9) | 34.5  (28.4 to 40.9) | 51.7  (49.1 to 54.3) | 83.7  (75.5 to 89.4) | 59.1  (54.3 to 63.9) |
| d-CPSS ≥4 | 82.0  (75.6 to 87.3) | 55.9  (49.3 to 62.3) | 58.8  (54.9 to 62.6) | 80.1  (74.4 to 84.6) | 67.2  (62.5 to 71.7) |
| d-CPSS ≥5 | 69.9  (62.7 to 76.5) | 75.2  (69.2 to 80.6) | 68.5  (63.0 to73.4) | 76.5  (72.1 to 80.4) | 72.9  (68.4 to 77.1) |
| d-CPSS ≥6 | 60.1  (52.6 to 67.3) | 83.6  (78.3 to 88.1) | 73.8  (67.4 to 79.4) | 73.2  (69.4 to 76.7) | 73.4  (68.9 to 77.6) |
| d-CPSS ≥7 | 47.5  (40.1 to 55.0) | 92.4  (88.3 to 95.5) | 82.9  (75.1 to 88.5) | 69.6  (66.5 to 72.6) | 72.9  (68.4 to 77.1) |
| d-CPSS ≥8 | 31.2  (24.5 to 38.4) | 97.5  (94.6 to 99.1) | 90.5  (80.7 to 95.6) | 64.8  (62.5 to 67.0) | 68.7  (64.0 to 73.1) |
| d-CPSS ≥9 | 14.8  (10.0 to 20.7) | 99.2  (97.0 to 99.9) | 93.1  (76.5 to 98.3) | 60.2  (58.7 to 61.7) | 62.5  (57.7 to 67.1) |
| d-CPSS =10 | 2.2  (0.6 to 5.5) | 99.6  (97.7 to 100.0) | 80.0  (31.1 to 97.3) | 57.0  (56.4 to 57.5) | 57.2  (52.4 to 62.0) |
| NIHSS ≥6^1^ | 87.4  (81.7 to 91.9) | 44.1  (37.7 to 50.7) | 54.6  (51.5 to 57.7) | 82.0  (75.2 to 87.3) | 63.0  (58.1 to 67.6) |
| NIHSS ≥8^1^ | 74.9  (67.9 to 81.0) | 64.7  (58.3 to 70.8) | 62.0  (57.4 to 66.4) | 77.0  (71.9 to 81.4) | 69.1  (64.5 to 73.5) |
| NIHSS ≥10^1^ | 66.7  (59.3 to 73.5) | 81.9  (76.5 to 86.6) | 73.9  (68.0 to 79.1) | 76.2  (72.1 to 79.8) | 75.3  (70.9 to 79.4) |
| NIHSS ≥11 | 64.5  (57.1 to 71.4) | 87.0  (82.0 to 91.0) | 79.2  (72.9 to 84.3) | 76.1  (72.3 to 79.6) | 77.2  (72.9 to 81.1) |

Abbreviation: CI, confidence interval; CPSS, Cincinnati Prehospital Stroke Scale; d-CPSS, detailed CPSS; NIHSS, National Institutes of Health Stroke Scale.

**Table S3** Baseline characteristics of the included and excluded patients

|  | Included patients  (N=421) | Excluded patients  (N=107) | P value |
| --- | --- | --- | --- |
| Age, years, median (IQR) | 68 (18) | 70 (15) | 0.110 |
| Gender, female, % (n) | 48.7 (205) | 46.7 (50) | 0.716 |
| NIHSS score, median (IQR) | 8 (9) | 5 (6) | **0.001** |
| Onset-to-assessment time, min, median (IQR) | 84 (71) | 85 (62) | 0.733 |
| On admission SBP, mmHg, mean (SD) | 164.0 (30.3) | 167.3 (28.6) | 0.311 |
| On admission DBP, mmHg, mean (SD) | 89.9 (16.7) | 90.9 (16.8) | 0.562 |
| Smoking, % (n), 57 missing | 33.8 (125) | 29.7 (30) | 0.439 |
| Hypertension, % (n), 19 missing | 79.6 (323) | 79.6 (82) | 0.990 |
| Diabetes mellitus, % (n), 24 missing | 23.1 (93) | 27.5 (28) | 0.362 |
| Hyperlipidaemia, % (n), 40 missing | 55.5 (213) | 63.5 (66) | 0.144 |
| Atrial fibrillation, % (n), 29 missing | 25.1 (100) | 17.8 (18) | 0.123 |
| Coronary artery disease, % (n), 35 missing | 21.2 (83) | 17.8 (18) | 0.457 |
| Chronic heart failure, % (n), 31 missing | 10.9 (43) | 13.9 (14) | 0.398 |

Abbreviation: LVO, large vessel occlusion; NIHSS, National Institutes of Health Stroke Scale; IQR, interquartile range; CPSS, Cincinnati Prehospital Stroke Scale; d-CPSS, detailed CPSS; SBP, systolic blood pressure; DBP, diastolic blood pressure; SD, standard deviation.
